# Risk areas and social determinants of health: a longitudinal epidemiological study with temporal analysis of visceral leishmaniasis in Mirandiba and Salgueiro, 2013-2022

**DOI:** 10.1590/S2237-96222026v35e20250292.en

**Published:** 2026-05-03

**Authors:** Luciano Lindolfo, Fabio Mathias Correa, Maurício C. Horta, Adriana Gradela

**Affiliations:** 1 Universidade Federal do Vale do São Francisco, UNVASF, Programa de Pós-Graduação em Ciências da Saúde e Biológicas, Petrolina, PE, Brazil Universidade Federal do Vale do São Francisco UNVASF Programa de Pós-Graduação em Ciências da Saúde e Biológicas Petrolina PE Brazil; 2 University of the Free State, Department f Mathematical Statistics and Actuarial Sciences, Bloemfontein, South Africa. University of the Free State Department of Mathematical Statistics and Actuarial Sciences Bloemfontein South Africa; 3 Universidade Federal do Vale do São Francisco, UNVASF, Colegiado de Medicina Veterinária, Petrolina, PE, Brazil. Universidade Federal do Vale do São Francisco UNVASF Colegiado de Medicina Veterinária Petrolina PE Brazil

**Keywords:** Risk Factors, Socioeconomic Factors, Incidence, Prevalence, Public Health Surveillance., Factores de Riesgo, Factores Socioeconómicos, Incidencia, Prevalencia, Vigilancia en Salud Pública.

## Abstract

**Objective:**

To describe risk areas and social determinants of visceral leishmaniasis in the municipalities of Salgueiro and Mirandiba, Pernambuco state, Brazil, focusing on guiding priority control and prevention actions.

**Methods:**

This was a longitudinal study with temporal analysis (2013-2022), using data from the Notifiable Health Conditions Information System. Annual and cumulative incidence rates, canine prevalence, human and canine censuses and social, environmental and economic vulnerabilities were analyzed. Correspondence analysis was used to explore associations between neighborhoods and vulnerabilities, and a logistic model was used to estimate risk of canine transmission.

**Results:**

Ninety-three human cases were reported, with an annual average of 2.5 in Mirandiba and 6.8 in Salgueiro, with a trend toward a gradual reduction in cases. The highest incidence rates occurred in 2015 and 2017 (Mirandiba: up to 53.0/100,000 inhabitants) and in 2014 and 2017 (Salgueiro: up to 26.9/100,000 inhabitants). The cumulative rate was higher in Mirandiba (16.4 versus 11.2/100,000 inhabitants). Most cases occurred in men (72.0% and 63.0%) and children aged 1-4 years (24.0% and 26.0%). Six neighborhoods, 35 blocks, and 457 households were visited. The dog-to-inhabitant ratio ranged from 0.14 to 0.21. Canine prevalence was higher in Salgueiro (21.4%) than in Mirandiba (9.5%), with estimated risk 100.0% higher. Vulnerabilities varied between neighborhoods and were associated with presence of garbage, debris, stray dogs, livestock and lack of urban infrastructure.

**Conclusions:**

Visceral leishmaniasis persists as a public health challenge, with transmission influenced by socio-environmental factors. The approach adopted allows us to identify priority areas and inform local policies.

## Resumen

**Objetivo:**

Describir las zonas de riesgo y los determinantes sociales de la leishmaniasis visceral en los municipios de Salgueiro y Mirandiba, Pernambuco, Brasil, con el objetivo de orientar las acciones prioritarias de control y prevención.

**Métodos:**

Estudio longitudinal con análisis temporal (2013-2022), utilizando datos del Sistema de Información de Enfermedades de Declaración Obligatoria. Se analizaron las tasas de incidencia anual y acumulada, la prevalencia canina, los censos humanos y caninos, y las vulnerabilidades socioambientales y económicas. Se utilizó un análisis de correspondencias para explorar las asociaciones entre los barrios y las vulnerabilidades, y se empleó un modelo logístico para estimar el riesgo de transmisión canina.

**Resultados:**

Se notificaron 93 casos humanos, con un promedio anual de 2,5 en Mirandiba y 6,8 en Salgueiro, con una tendencia a la reducción gradual de los casos. Las tasas de incidencia más altas se registraron en 2015 y 2017 (Mirandiba: hasta 53,0/100.000 habitantes) y en 2014 y 2017 (Salgueiro: hasta 26,9/100.000 habitantes). La tasa acumulada fue mayor en Mirandiba (16,4 frente a 11,2/100.000 habitantes). La mayoría de los casos ocurrieron en hombres (72,0% y 63,0%) y niños de 1 a 4 años (24,0% y 26,0%). Se visitaron seis barrios, 35 manzanas y 457 hogares. La razón perro-habitante osciló entre 0,14 y 0,21. La prevalencia canina fue mayor en Salgueiro (21,4%) que en Mirandiba (9,5%), con un riesgo estimado 100,0% mayor. Las vulnerabilidades variaron entre barrios y se asociaron con la presencia de basura, escombros, perros callejeros, ganado y la falta de infraestructura urbana.

**Conclusiones:**

La leishmaniasis visceral persiste como un desafío de salud pública, con transmisión influenciada por factores socioambientales. El enfoque adoptado nos permite identificar áreas prioritarias e informar políticas locales.

**Ethical aspects**

This research respected ethical principles, having obtained the following approval data:

Research ethics committee: Autarquia Educacional de Belo Jardim

Opinion number: 6,022,569

Approval date: 7/3/2023

Certificate of submission for ethical appraisal: 60397922.0.0000.5189

Informed consent record: Obtained from all participants prior to data collection.

## Introduction 

Visceral leishmaniasis is a systemic, non-contagious infectious protozoan disease that affects humans and other animals. Transmission of visceral leishmaniasis occurs primarily through the bite of the female insect vector *Lutzomyia longipalpis*, commonly known as the sandfly. Classified as the second most prevalent protozoan disease worldwide, it represents a serious public health problem in five regions: the Americas, East Africa, North Africa, West Asia, and Southeast Asia ^1^. In 2019, within the Americas, Brazil accounted for 97.0% of cases, with prevalence across all the country's regions, with the Northeast region accounting for approximately 90.0% of reported cases ^2^.

The transmission patterns of visceral leishmaniasis in Brazil have changed over the years, with cases emerging in urban and peri-urban areas. These changes in the epidemiological patterns of the disease involve the coexistence of different vector species, reservoirs and etiological agents, associated with anthropogenic environmental changes, posing a challenge for planning effective control actions ^3^.

In Northeastern Brazil, despite the actions implemented by the Visceral Leishmaniasis Surveillance and Control Program, the disease remains highly prevalent and fatal, with its control being complex due to the multiple social determinants of health associated with its transmission. These determinants favor increased sandfly density, vector proliferation and the establishment of a transmission chain conducive to the disease ^4^. Promising associations have been identified between spatial patterns of morbidity and mortality and socioeconomic and environmental factors, suggesting the relevance of incorporating these factors into prevention and control strategies ^5^.

Effective control of visceral leishmaniasis requires greater integration of surveillance, personal protection, environmental management and health education activities. Such integration should be coupled with an assessment of the spatial distribution of risk and health vulnerabilities in high-incidence municipalities, aiming to establish priorities and specific strategies for health surveillance.

This study aimed to describe the risk areas and social determinants of health related to the transmission of visceral leishmaniasis in Salgueiro and Mirandiba, in the state of Pernambuco, with the aim of establishing the order of priority for prevention and control actions.

## Methods 

Design

This was an observational epidemiological study of a longitudinal nature and with temporal analysis that investigated the occurrence of cases of visceral leishmaniasis in Mirandiba and Salgueiro between January 2013 and December 2022. The data used were extracted from the Notifiable Health Conditions Information System.

Setting

The municipalities of Mirandiba and Salgueiro, 63 km apart, are located in the Pernambuco backlands, or *sertão* in Portuguese. Mirandiba has a territorial area of 821.676 km² and, in 2023, its population was estimated at 14,166 inhabitants, it had a population density of 17.24 inhabitants/km² and its municipal human development index was 0.591 ^6^. Salgueiro has a territorial area of 1,678.564 km² and, in 2023, it had a population density of 37.16 inhabitants/km² and its municipal human development index was 0.669 ^6^.

Participants

This study analyzed confirmed cases of visceral leishmaniasis in Mirandiba and Salgueiro between January 2013 and December 2022, extracted from the Notifiable Health Conditions Information System. Prior to analysis, the records were processed to eliminate inconsistencies and duplications, so as to ensure data quality. The extent of missing data in primary variables (sex, age, municipality of residence, etc.) was quantified as part of the assessment of record completeness.

Assessment of health vulnerability was conducted using a form developed specifically for this study, concerning households located in neighborhoods with a higher probability of visceral leishmaniasis transmission. A census of dogs and human beings was conducted in the same households to estimate the dog-to-inhabitant ratio.

Prevalence of canine visceral leishmaniasis during the same period was estimated based on data from the Laboratory Environment Management System.

Data on the resident population in the municipalities were obtained from projections made by the Brazilian Institute of Geography and Statistics, accessed via TabNet.

Variables

Using data extracted from the Notifiable Health Conditions Information System, we obtained information on sex, age group, year of notification, mode of admission (new case), final classification (confirmed) and classification criteria (laboratory or clinical-epidemiological). The annual and cumulative incidence rates were calculated by dividing the total number of new cases multiplied by 100,000 by the population of the year and period, respectively. The municipalities were classified according to visceral leishmaniasis, transmission, following the recommendations of the Endemic Control Department: sporadic transmission (average annual cases <2.4), moderate transmission (average annual cases ≥2.4 and <4.4) and intense transmission (average annual cases ≥4.4) ^7^.

Risk of visceral leishmaniasis transmission was estimated by dividing the municipalities into neighborhoods, according to the Family Health Strategy methodology, to mitigate potential selection bias and increase representation of the municipalities. Risk classification considered case frequency in the 2019-2022 period, the incidence rate, and the dog-to-inhabitant ratio. Case frequency in each neighborhood was classified as low (1-2 years), medium (3 years) or high (>4 years), while the incidence rate was classified as low (lower than the average cumulative incidence rate) or high (higher than the average cumulative incidence rate). The dog-to-inhabitant ratio was estimated based on a census of dogs and human beings conducted in these neighborhoods.

The definition of the sample of blocks and properties, based on the universe of properties and blocks (active and inactive) per neighborhood provided by the Locality Registration System, employed a methodology adapted from the Rapid *Aedes aegypti* Indices Survey ^8^, with the objective of maximizing the accuracy of the estimate within the cost constraints. In each selected block, 50.0% of the households were systematically visited in clockwise order (first yes, second no, third yes, and subsequent in case of empty households) until the sample was complete.

In order to analyze health vulnerabilities regarding visceral leishmaniasis transmission, three categories (social, economic and environmental) and 25 questions (Q) were established. Social vulnerability was assessed according to: unpaved streets (Q1), exposure to open sewer (Q2), accumulated domestic waste (Q3) or accumulation of other types of garbage (Q4), piles of earth and/or organic material (Q5), piles of wood, leaves etc. (Q6), waste land (Q7), stray dogs roaming the streets (Q8) and individual or collective kennels (Q9). Economic vulnerability was assessed based on presence of home built of masonry (Q10), external area of home rendered (Q11), presence of pavement (Q12), backyard with concrete floor (Q13), backyard without walls or with wooden fencing (Q14), backyard with a dirt floor (Q15), garbage or debris in backyard (Q16), backyard damp (Q17), plants in the backyard (Q18). Environmental vulnerability was assessed based on presence of tall vegetation (Q19) or undergrowth (Q20), lakes or ponds of water (Q21), dampness due to lack of basic sanitation (Q22), livestock raising (Q23), chicken coops (Q24) and pig pens or corrals (Q25). 

In order to mitigate subjectivity in assessing health vulnerabilities, structured checklists were developed with defined indicators for each variable. The presence or absence of each indicator was recorded, thus standardizing observation. A sample of the observed conditions was also photographically documented.

Data processing and analysis 

The data were processed and tabulated using TabWin32 and Microsoft Excel 365 software. Statistical analysis included calculation of absolute and relative distributions, as well as correspondence analysis to investigate associations between municipalities/neighborhoods and vulnerability variables. Binary logistic regression was applied to estimate association between municipality of residence (Salgueiro vs. Mirandiba) and probability of canine visceral leishmaniasis transmission. Canine positivity (1 = positive; 0 = negative) was taken as the dependent variable, while municipality (1 = Salgueiro; 0 = Mirandiba) was taken as the explanatory variable. All statistical analyses were conducted using R software version 4.3.3, supported by the FactoMineR and Factoextra packages ^9^^,^^10^.

## Results 

Between January 2013 and December 2022, 93 new visceral leishmaniasis cases were reported in Mirandiba and Salgueiro. Of these, 26.8% (n=25) occurred in Mirandiba and 73.2% (n=68) in Salgueiro. The annual average number of cases was 2.5 in Mirandiba and 6.8 in Salgueiro. The incidence rates ranged from 0-53.0 cases per 100,000 inhabitants in Mirandiba and from 1.6-26.9 cases per 100,000 inhabitants in Salgueiro. The highest incidence rates in Mirandiba were observed in 2015 (53.0) and 2017 (26.2) and in Salgueiro in 2014 (26.9) and 2017 (21.5) (Figure 1). Although Salgueiro had the highest number of cases, the cumulative incidence rate in the period was higher in Mirandiba (16.4 vs. 11.2).

**Figure 1:**
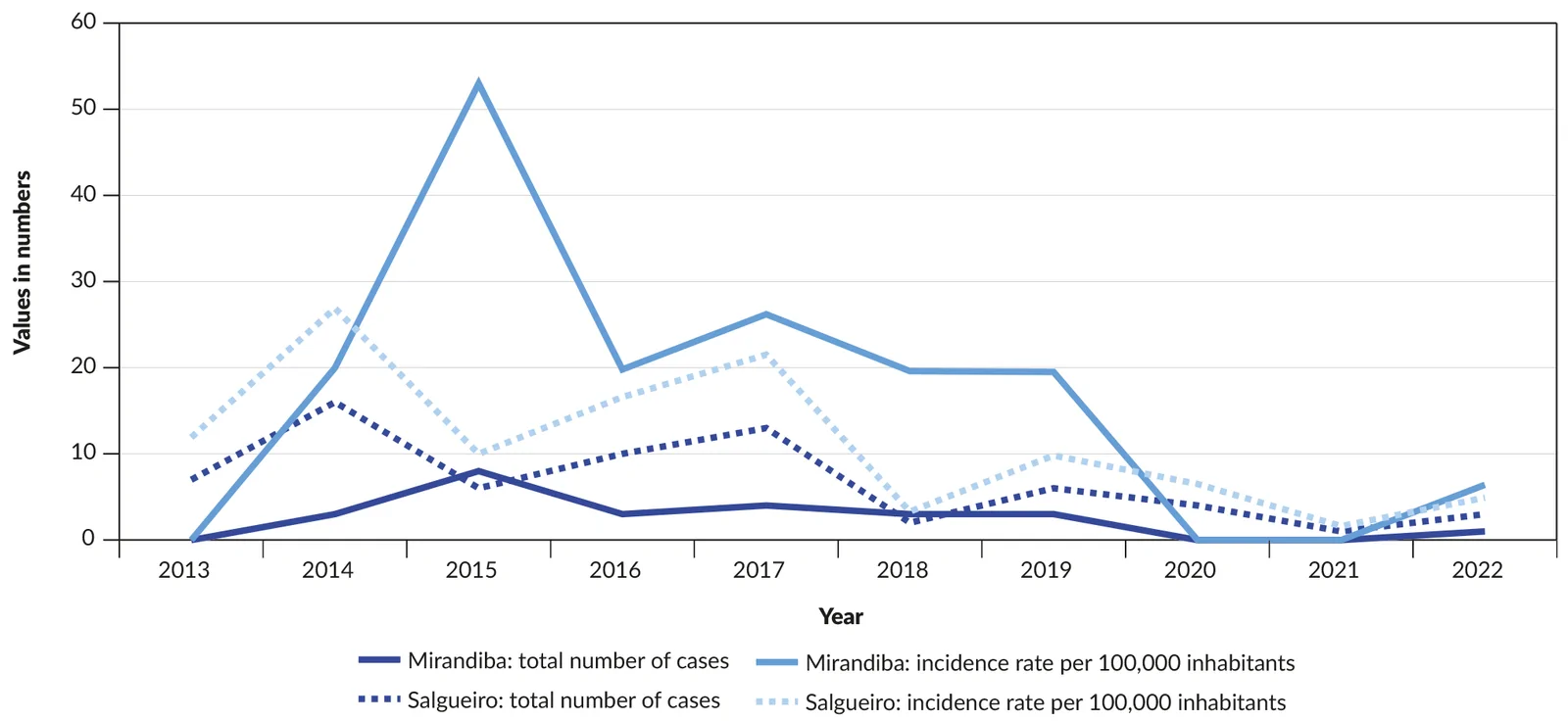
Total visceral leishmaniasis cases and incidence rate per 100,000 inhabitants. Mirandiba and Salgueiro, 2013-2022

Visceral leishmaniasis presented similar sociodemographic characteristics in both municipalities, with a higher frequency in males (n=18 in Mirandiba and n=43 in Salgueiro), corresponding to 72.0% and 63.0% of cases. The most affected age group was 1-4 years (n=6 in Mirandiba and n=18 in Salgueiro), representing 24.0% and 27.0% of cases.

In Mirandiba, 100.0% of the neighborhoods listed in the Family Health Strategy (n=2: Cohab and Projetada) presented cases of visceral leishmaniasis in the period 2019-2022 and were included in the risk analysis. In Salgueiro, of the 14 neighborhoods listed, only 29.0% were included (n=4: Barriguda, Divino, Planalto and Prado), accounting for 50.0% of the cases reported in the period (n=7/14). Case frequency in consecutive years was considered low (1-2 cases) in all neighborhoods, classifying them as having low risk of transmission. However, Prado and Cohab were classified as high risk based on the average cumulative incidence rate, which was higher than the overall incidence rate (Table 1).

In Mirandiba, 15 blocks were selected and 217 households were visited, distributed between the Cohab neighborhood (8 blocks and 112 households) and the Projetada neighborhood (7 blocks and 105 households). In the Cohab neighborhood, the dog-to-inhabitant ratio was 0.15 (52 dogs / 353 inhabitants), while in Projetada it was 0.14 (49 dogs / 360 inhabitants). In Salgueiro, 20 blocks were selected and 240 households were visited, with the following distribution by neighborhood: Barriguda (2 blocks and 30 households), Divino (3 blocks and 63 households), Planalto (12 blocks and 47 households) and Prado (3 blocks and 100 households). The dog/inhabitant ratio was 0.14 (16 dogs / 117 inhabitants) in Barriguda, 0.15 (29 dogs / 199 inhabitants) in Divino, 0.21 (36 dogs / 174 inhabitants) in Planalto, and 0.14 (42 dogs / 293 inhabitants) in Prado. The order of priority for control actions, established based on the risk classification (Table 1), and the spatial distribution of risk areas in the two municipalities (Figure 2) were obtained.

**Table 1:** Order of priority for control actions based on case frequency, incidence rate (IR) per 100,000 inhabitants and dogs/inhabitant ratio (DIR). Mirandiba and Salgueiro, 2019-2022

Municipality	Neighborhood	Case frequency	CCF^a^	Population in 2022	IR	CAIR^b^	CIR^c^	DIR	Order of priority
Mirandiba	Cohab	1	Low	3,153	7.90	6.55	High	0.15	1
	Projetada	1	Low	4,815	5.20	-	Low	0.14	2
	Total	2	-	7,968	-	-	-	0.14	-
Salgueiro	Barriguda	1	Low	2,298	10.90	11.72	Low	0.14	4
	Divino	2	Low	5,042	9.90	-	Low	0.15	3
	Planalto	2	Low	4,663	10.70	-	Low	0.21	2
	Prado	2	Low	3,253	15.40	-	High	0.14	1
	Total	7	-	15,256	-	-	-	0.16	-

a Classification according to case frequency; ^b^Cumulative average incidence rate per 100,000 inhabitants; ^c^Classification according to incidence rate.

**Figure 2:**
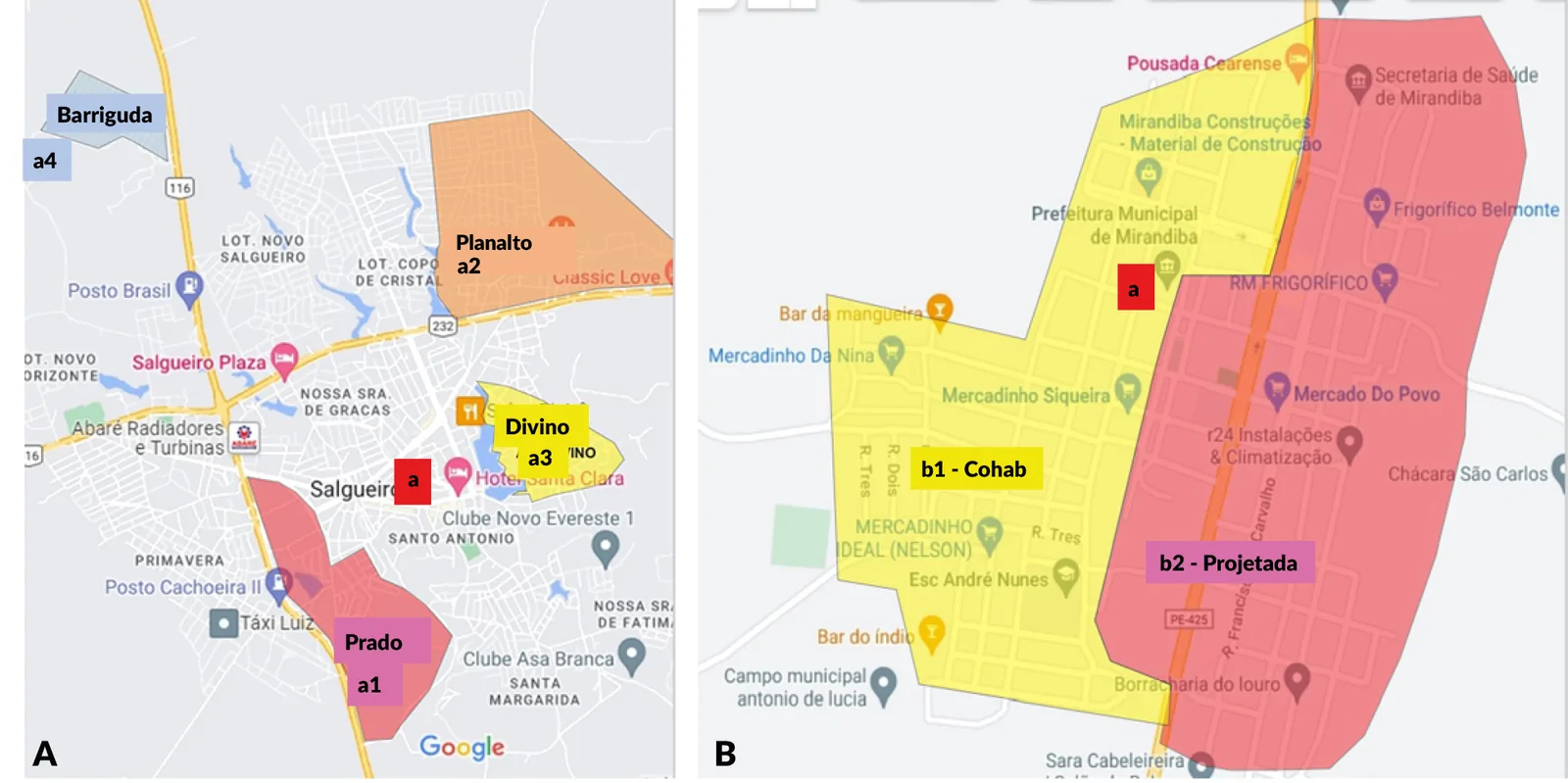
Spatial distribution of visceral leishmaniasis risk areas by neighborhood. Salgueiro (A) and Mirandiba (B), 2022 ^a^City Hall; ^b^Order of priority for control actions (1 - higher and 4 - lower).

**Figure 3:**
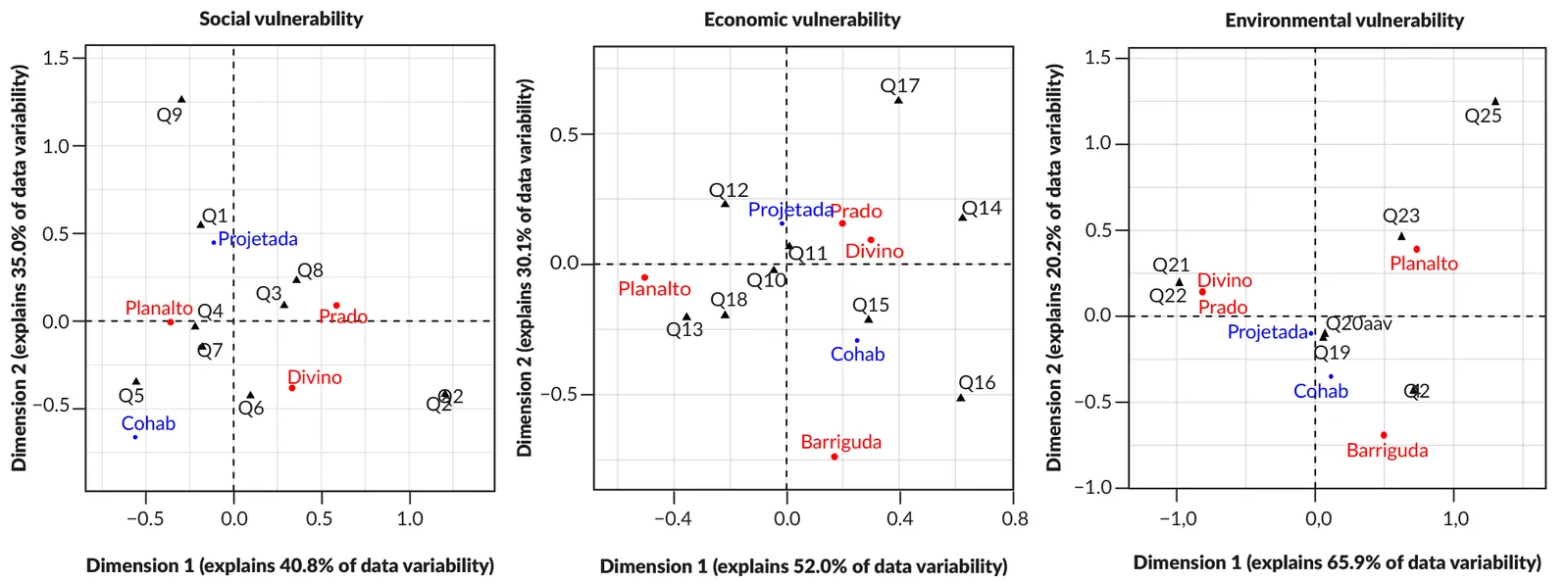
Correspondence analysis by type of vulnerability: (A) social (Q1-Q9), (B) economic (Q10-Q18) and (C) environmental (Q19-Q25) in the neighborhoods of the municipalities studied. Mirandiba (blue) and Salgueiro (red), 2022 ^Q1^Unpaved streets, ^Q2^Exposure to open sewers, ^Q3^Accumulated household waste, ^Q4^Accumulation of other types of garbage, ^Q5^Presence of piles of earth and/or organic material, ^Q6^Piles of wood, leaves etc., ^Q7^Waste land, ^Q8^Stray dogs roaming the streets, ^Q9^Individual or collective kennels, ^Q10^Home built with masonry, ^Q11^External area of home rendered, ^Q12^Presence of pavement, ^Q13^Concrete backyard, ^Q14^Backyard with no walls or with wooden fence, ^Q15^Backyard with a dirt floor, ^Q16^Garbage or debris in backyard, ^Q17^Backyard damp, ^Q18^Plants in the backyard, ^Q19^Presence of tall vegetation, ^Q20^Presence of undergrowth, ^Q21^Lakes or ponds of water, ^Q22^Dampness due to lack of basic sanitation, ^Q23^Livestock raising, ^Q24^Chicken coops and ^Q25^Pig pens or corrals.

Correspondence analysis suggested differences in the patterns of social, economic and environmental vulnerability between the neighborhoods of Salgueiro and Mirandiba, as different associations were observed in each neighborhood (Figure 3). Vulnerabilities were relatively dispersed, indicating that different neighborhoods may be associated with different sets of vulnerabilities or a single set of vulnerabilities.

The social vulnerability graph (Figure 3A) presented two dimensions: dimension 1 explained 40.8% of the total variance, and dimension 2, 35.0% of the total variance. In Mirandiba, social vulnerabilities were associated with the presence of debris (Q5) in the Cohab neighborhood. In the Projetada neighborhood, social vulnerabilities were associated with unpaved streets (Q1), presence of domestic waste (Q3) and other types of garbage (Q4), stray dogs (Q8) and individual or collective kennels (Q9). In Salgueiro, there was no significant association in the Barriguda neighborhood. On the other hand, the Divino neighborhood was associated with the presence of piles of wood and leaves (Q6) and stray dogs (Q8); the Planalto neighborhood was associated with the presence of other types of garbage (Q4), waste land (Q7) and stray dogs roaming the streets (Q8); the Prado neighborhood was associated with accumulated domestic waste (Q3) and stray dogs roaming the streets (Q8). 

In the economic vulnerability graph (Figure 3B), dimension 1 explained 52.0% of the total variance, and dimension 2, 30.1%. In Mirandiba, economic vulnerabilities were associated with the presence of a backyard with a dirt floor (Q15) in the Cohab neighborhood and external area of home rendered (Q11) in the Projetada neighborhood. In Salgueiro, the Barriguda neighborhood was associated with presence of garbage or debris in the backyard (Q16); the Divino neighborhood was associated with external area of home rendered (Q11), backyard without walls or with wooden fencing (Q14) and backyard with a dirt floor (Q15); the Planalto neighborhood was associated with backyards with a concrete floor (Q13) and backyards with presence of plants (Q18); the Prado neighborhood was associated with external area of home rendered (Q11) and backyard without walls or with wooden fencing (Q14).

In the environmental vulnerability graph (Figure 3C), dimensions 1 and 2 explained 65.9% and 20.2% of the total variance. In Mirandiba, environmental vulnerabilities were associated with the presence of tall vegetation (Q19) in the Cohab and Projetada neighborhoods, and the Projetada neighborhood also presented association with undergrowth (Q20). In Salgueiro, the Barriguda neighborhood was associated with chicken coops (Q24); the Divino and Prado neighborhoods were associated with presence of lakes or ponds (Q21), dampness due to lack of basic sanitation (Q22) and presence of livestock raising (Q23); while the Planalto neighborhood was associated with livestock raising (Q23) and presence of pig pens or corrals (Q25). 

Canine visceral leishmaniasis prevalence was significantly higher (p-value=0.99) in Salgueiro (21.4%) than in Mirandiba (9.5%). In Salgueiro, positivity ranged from 6.0% to 55.0%, and negativity from 45.0% to 94.0%. The highest positivity was in 2015, and the highest negativity in 2018. In Mirandiba, positivity ranged from 0.0% to 49.0%, and negativity from 0.0% to 100.0%. The highest positivity was in 2015, and the highest negativity in 2013 and 2020.

The logistic model, which took Mirandiba as the reference category (odds ratio, OR 1.0), showed that dogs living in Salgueiro were twice as likely to be seropositive for visceral leishmaniasis compared to dogs in Mirandiba (OR 2.00; 95% confidence interval 1.32; 3.03; p-value=0.001). This finding indicated that, even controlling for temporal variability, Salgueiro presented a significantly higher risk, justifying its classification as a priority area for surveillance and control actions.

The trend graph (Figure 4) demonstrated that, despite the reduction in rates over the years, the estimated probability of canine positivity remained consistently higher in Salgueiro. The confidence intervals highlighted in the curve (green shaded areas) reinforced the robustness of the estimate.

**Figure 4:**
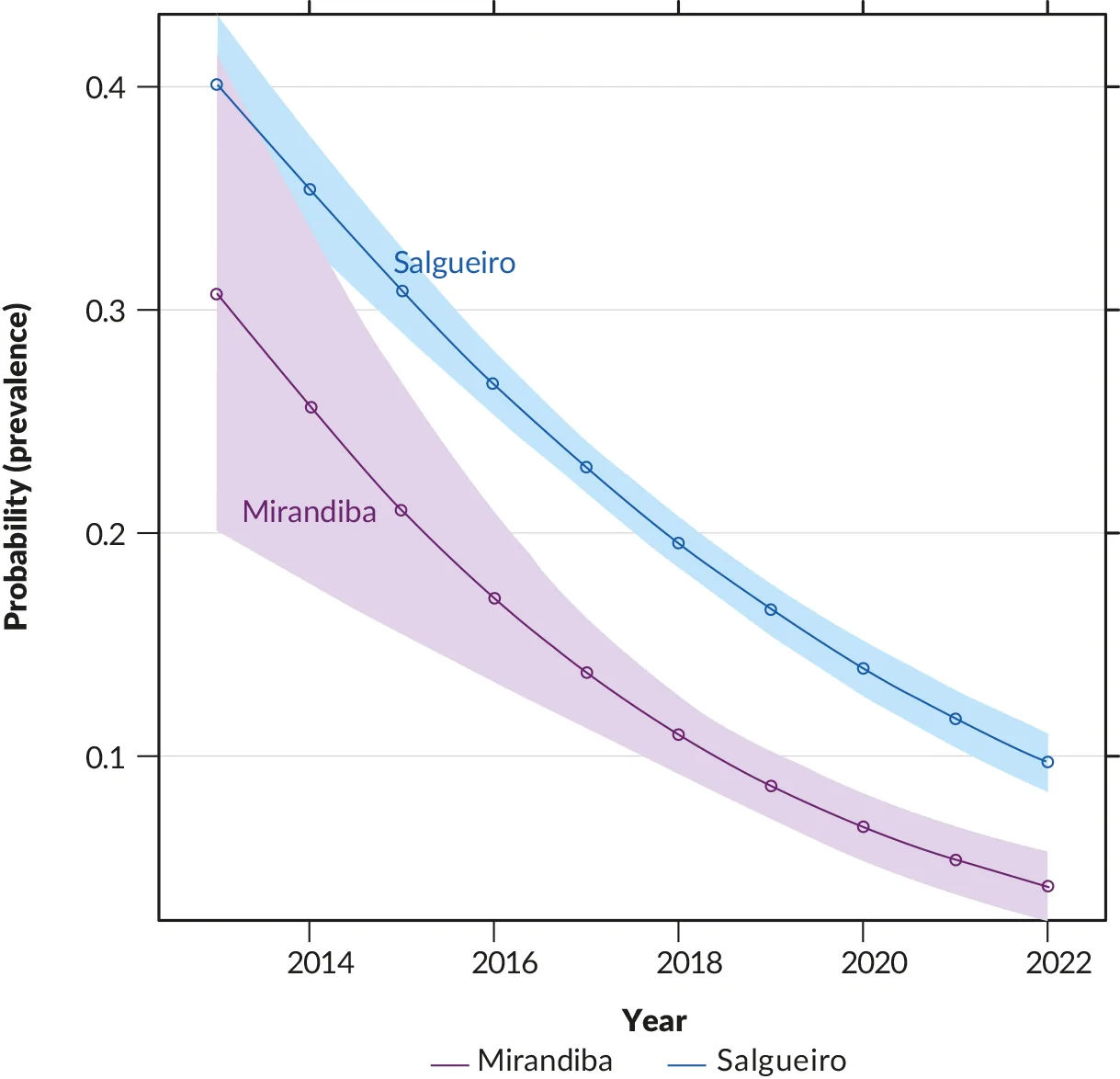
Prevalence of canine visceral leishmaniasis. Salgueiro and Mirandiba, 2013-2022

## Discussion 

Visceral leishmaniasis continues to be a public health problem in Mirandiba and Salgueiro, both classified as areas of intense transmission ^7^, with incidence rates higher than the national and state averages ^11^^,^^12^, even after the falling trend observed since 2017. The findings demonstrate that the persistence of transmission is linked to structural factors, such as poverty, poor sanitation, inadequate solid waste disposal ^13^, unplanned urbanization ^11^ and high prevalence of infected canine reservoirs ^14^^,^^15^.

Higher occurrence of visceral leishmaniasis in males, consistent with previous findings ^5^^,^^16^, may be related to hormonal factors, greater occupational exposure and mobility patterns that coincide with the vector's feeding schedules ^16^. This profile is especially relevant in regions where agriculture predominates as the main source of income ^12^, as in the municipalities studied.

High incidence in children aged 1-4 years, also observed in other studies ^5^^,^^11^^,^^17^, highlights the role of transmission in the home and peridomestic environment. Immunological susceptibility associated with an immature immune system and the presence of nutritional deficiencies ^18^, combined with interaction with domestic animals ^19^, contributes to increased risk in this age group. These findings reinforce the need for intersectoral strategies aimed at improving nutritional status ^18^, canine population control and active surveillance.

Multivariate analysis identified distinct territorial vulnerability profiles, such as garbage accumulation, debris, dense vegetation, livestock, and stray dogs ^20^^,^^21^^,^^22^^,^^23^. These findings contribute to understanding the dynamics of the urbanization of this disease and support territorial planning for surveillance and control actions.

Territories for visceral leishmaniasis control were prioritized based on multiple epidemiological and environmental criteria, including human case frequency, average cumulative incidence rate, proportion of affected neighborhoods, dog-to-inhabitant ratio, and prevalence of seropositive dogs-a widely recommended approach for delimiting risk areas in urban disease transmission scenarios ^5^^,^^17^. The integrated application of these indicators allowed the territories studied to be classified into low-, medium-, and high-risk areas, thus guiding more targeted surveillance and control actions.

For example, in Mirandiba, the Cohab neighborhood had cumulative incidence higher than the municipal average, a dog-to-inhabitant ratio above 0.14, and multiple structural vulnerabilities, justifying its inclusion as a high-priority area. In Salgueiro, the Prado and Planalto neighborhoods concentrated simultaneous epidemiological and environmental risk factors, such as high canine prevalence, stray dogs roaming the streets, and environmental vulnerabilities (wasteland, accumulated garbage and livestock raising), these being factors that favor the persistence of urban vector ecotopes ^22^^,^^23^^,^^24^.

High prevalence of canine visceral leishmaniasis in Salgueiro (21.4%), combined with the presence of asymptomatic seropositive dogs capable of infecting the vector (25.26), resulted in estimated risk 100.0% higher than that of Mirandiba. This reinforced the importance of combining epidemiological and environmental parameters for defining territories ^5^.

Correspondence analysis confirmed that vulnerabilities are not evenly distributed, but rather vary between neighborhoods and municipalities. In Mirandiba, association was observed with unpaved streets, household waste, yards with accumulated waste and the presence of makeshift kennels-conditions that favor the proliferation of sand flies ^24^^,^^25^^,^^26^. In Salgueiro, the proximity of chicken coops, ponds, wasteland and damp areas due to lack of basic sanitation stood out, these being factors directly associated with increased vector density ^14^^,^^22^^,^^23^.

These findings reinforce the need for territorial prioritization to adopt a multidimensional model, incorporating epidemiological, social, environmental and structural factors, in accordance with established guidelines ^27^ and scientific evidence ^5^^,^^22^^,^^27^. This approach contributes to the efficient allocation of resources, the planning of integrated actions, as well as the strengthening of local public policies.

Although the findings of this study are relevant, some methodological limitations should be considered. Neighborhood selection was based on operational and logistical criteria, with inclusion restricted to areas covered by Family Health Strategy teams and with a history of reported cases, which may have compromised territorial representativeness and led to overestimation or underestimation of risk in certain locations. The absence of random selection and probabilistic sampling limited the extrapolation of results to other neighborhoods within the municipalities. Underreporting of human cases during the COVID-19 pandemic, particularly in 2020 and 2021, also impacted the temporal analysis, reducing the accuracy of incidence estimates and hindering identification of epidemiological trends ^17^. The lack of data on vector density prevented more robust correlations between environmental factors and transmission dynamics, limiting understanding of the role of sandflies in maintaining the disease cycle.

These limitations reinforce the need for future studies with a broader territorial scope, systematic entomological monitoring and assessment of the effects of the pandemic on surveillance indicators. Nevertheless, the results presented offer relevant insights for municipal planning, especially regarding the delimitation of priority areas.

Integrated and sustainable strategies that combine health, environment, infrastructure and education are recommended to address visceral leishmaniasis in urban and vulnerable contexts. Adopting territorial criteria in planning allows resources to be allocated according to epidemiological and environmental risk, with actions focused on priority neighborhoods, such as those identified in this study.

In addition to epidemiological and entomological surveillance, it is essential to strengthen early diagnosis, monitor the canine population with appropriate sanitary measures and perform environmental control of vector breeding sites. Community participation should be encouraged through health education, awareness campaigns on waste management and safe coexistence with domestic animals. Such actions must be continuous and coordinated with sanitation, social protection and sustainable urban development policies.

The results obtained offer technical support for the formulation of territorialized, equitable and evidence-based public policies, essential to reduce the burden of visceral leishmaniasis and strengthen control in municipalities with high socio-environmental vulnerability.

## Data Availability

The data are available at: https://zenodo.org/records/15933982.
