# Supplementary material for: Risk areas and social determinants of health: a longitudinal epidemiological study with temporal analysis of visceral leishmaniasis in Mirandiba and Salgueiro, 2013-2022
Source: Epidemiol Serv Saude. 2026 May 3;35:e20250292. doi: 10.1590/S2237-96222026v35e20250292.en (PMC13686424; doi:10.1590/S2237-96222026v35e20250292.en)
Supplement: Translation

# Translation: Áreas de risco e determinantes sociais da saúde: estudo epidemiológico longitudinal com análise temporal da leishmaniose visceral em Mirandiba e Salgueiro, 2013-2022

**DOI:** 10.1590/S2237-96222026v35e20250292.pt

**Authors:** Luciano Lindolfo, Fabio Mathias Correa, Maurício C. Horta, Adriana Gradela

**Affiliations:** 1 Universidade Federal do Vale do São Francisco, UNVASF, Programa de Pós-Graduação em Ciências da Saúde e Biológicas, Petrolina, PE, Brasil Universidade Federal do Vale do São Francisco UNVASF Programa de Pós-Graduação em Ciências da Saúde e Biológicas Petrolina PE Brasil; 2 University of the Free State, Department f Mathematical Statistics and Actuarial Sciences, Bloemfontein, South Africa. University of the Free State Department of Mathematical Statistics and Actuarial Sciences; 3 Universidade Federal do Vale do São Francisco, UNVASF, Colegiado de Medicina Veterinária, Petrolina, PE, Brasil. Universidade Federal do Vale do São Francisco UNVASF Colegiado de Medicina Veterinária Petrolina PE Brasil

**Keywords:** Fatores de Risco, Fatores Socioeconômicos, Incidência, Prevalência, Vigilância em Saúde Pública.

## Abstract

**Objetivo:**

Descrever áreas de risco e determinantes sociais da leishmaniose visceral nos municípios pernambucanos Salgueiro e Mirandiba, com foco na orientação de ações prioritárias de controle e prevenção.

**Métodos:**

Estudo longitudinal com análise temporal (2013-2022), utilizando dados do Sistema de Informação de Agravos de Notificação. Foram analisadas taxas de incidência anual e acumulada, prevalência canina, censos humano e canino e vulnerabilidades socioambientais e econômicas. Aplicaram-se análise de correspondência para explorar associações entre bairros e vulnerabilidades e modelo logístico para estimar o risco de transmissão canina.** **

**Resultados:**

Foram notificados 93 casos humanos, com média anual de 2,5 em Mirandiba e 6,8 em Salgueiro, com tendência de redução gradual dos casos. As maiores taxas de incidência ocorreram em 2015 e 2017 (Mirandiba: até 53,0/100 mil habitantes) e em 2014 e 2017 (Salgueiro: até 26,9/100 mil habitantes). A taxa acumulada foi maior em Mirandiba (16,4 versus 11,2/100 mil habitantes). A maioria dos casos ocorreu em homens (72,0% e 63,0%) e crianças de 1-4 anos (24,0% e 26,0%). Foram visitados seis bairros, 35 quarteirões e 457 domicílios. A razão cães/habitante variou de 0,14 a 0,21. A prevalência canina foi maior em Salgueiro (21,4%) do que em Mirandiba (9,5%), com risco estimado 100,0% superior. As vulnerabilidades variaram entre bairros, associando-se à presença de lixo, entulhos, cães errantes, criação de animais e à ausência de infraestrutura urbana.

**Conclusões:**

A leishmaniose visceral persiste como desafio de saúde pública, com transmissão influenciada por fatores socioambientais. A abordagem adotada permite identificar áreas prioritárias e subsidiar políticas locais.

**Aspectos éticos**

Esta pesquisa respeitou os princípios éticos, obtendo os seguintes dados de aprovação:

Comitê de ética em pesquisa: Autarquia Educacional de Belo Jardim

Número do parecer: 6.022.569

Data de aprovação: 7/3/2023

Certificado de apresentação de apreciação ética: 60397922.0.0000.5189

Registro do consentimento livre e esclarecido: Obtido de todos os participantes antes da coleta.

## Introdução 

A leishmaniose visceral é uma protozoose infecciosa sistêmica e não contagiosa que acomete humanos e outros animais. A transmissão da leishmaniose visceral ocorre principalmente através da picada da fêmea do inseto vetor *Lutzomyia longipalpis*, cujo nome popular é mosquito palha. Classificada como a segunda protozoose mais prevalente no mundo, representa um grave problema de saúde pública em cinco regiões: Américas, África Oriental, Norte da África, Ásia Ocidental e Sudeste Asiático ^1^. Em 2019, no contexto das Américas, o Brasil concentrava 97,0% dos casos, com prevalência em todas as suas regiões, destacando-se o Nordeste, responsável por aproximadamente 90,0% das notificações ^2^.

Os padrões de transmissão da leishmaniose visceral no Brasil têm apresentado modificações ao longo dos anos, com a emergência de casos em áreas urbanas e periurbanas. Essas mudanças nos padrões epidemiológicos da doença envolvem a coexistência de diferentes espécies de vetores, reservatórios e agentes etiológicos, associada a alterações ambientais antropogênicas, configurando-a como um desafio para o planejamento de ações de controle eficazes ^3^.

No Nordeste brasileiro, apesar das ações implementadas pelo Programa de Vigilância e Controle da Leishmaniose Visceral, a endemia mantém alta incidência e letalidade, com controle complexo devido à multiplicidade de determinantes sociais de saúde relacionados à sua transmissão. Esses determinantes favorecem o aumento da densidade de flebotomíneos, a proliferação vetorial e o estabelecimento de uma cadeia de transmissão favorável à doença ^4^. Identificaram-se associações promissoras entre padrões espaciais de morbimortalidade e fatores socioeconômicos e ambientais, sugerindo a relevância da incorporação desses fatores nas estratégias de prevenção e controle ^5^.

O controle efetivo da leishmaniose visceral exige maior integração das atividades de vigilância, proteção individual, gestão ambiental e educação em saúde. Essa integração deve estar associada à avaliação da distribuição espacial do risco e das vulnerabilidades de saúde em municípios de alta incidência, visando ao estabelecimento de prioridades e estratégias específicas pela vigilância em saúde.

Este estudo objetivou descrever as áreas de risco e os determinantes sociais de saúde relacionados à transmissão da leishmaniose visceral em Salgueiro e Mirandiba, em Pernambuco, com o intuito de estabelecer a ordem de prioridade das ações de prevenção e controle.

## Métodos 

Delineamento do estudo

Tratou-se de estudo epidemiológico observacional de natureza longitudinal e com análise temporal que investigou a ocorrência de casos de leishmaniose visceral em Mirandiba e Salgueiro entre janeiro de 2013 e dezembro de 2022. Os dados utilizados foram extraídos do Sistema de Informação de Agravos de Notificação.

Contexto do estudo

Os municípios de Mirandiba e Salgueiro, distantes 63 km entre si, estão localizados no sertão pernambucano. Mirandiba possui área territorial de 821,676 km² e, em 2023, a população foi estimada em 14.166 habitantes, a densidade demográfica era 17,24 habitantes/km² e o índice de desenvolvimento humano municipal, 0,591 ^6^. Salgueiro apresenta área territorial de 1.678,564 km² e, em 2023, a densidade demográfica era 37,16 habitantes/km² e o índice de desenvolvimento humano municipal, 0,669 ^6^.

Participantes do estudo

Este estudo analisou os casos confirmados de leishmaniose visceral em Mirandiba e Salgueiro entre janeiro de 2013 e dezembro de 2022, extraídos do Sistema de Informação de Agravos de Notificação. Previamente à análise, os registros foram processados para eliminar inconsistências e duplicidades, assegurando a qualidade das informações. A extensão dos dados faltantes nas variáveis primárias (sexo, idade, município de residência etc.) foi quantificada como parte da avaliação da completude dos registros.

A avaliação das vulnerabilidades de saúde foi conduzida através da aplicação de formulário elaborado especificamente para este estudo, direcionado aos domicílios situados nos bairros com maior probabilidade de transmissão de leishmaniose visceral. Nesses mesmos endereços, realizaram-se um censo canino e um censo humano para estimar a razão cães/habitante. 

A prevalência de leishmaniose visceral canina no mesmo período foi estimada a partir de dados do Gerenciador de Ambiente Laboratorial.

Os dados de população residente nos municípios foram obtidos das projeções do Instituto Brasileiro de Geografia e Estatística, acessadas através do TabNet.

Variáveis analisadas

A partir dos dados extraídos do Sistema de Informação de Agravos de Notificação, foram obtidas as informações sobre sexo, faixa etária, ano de notificação, modo de entrada (caso novo), classificação final (confirmado) e critério de classificação (laboratorial ou clínico-epidemiológico). As taxas de incidência anual e acumulada foram calculadas dividindo-se o número total de casos novos multiplicados por 100 mil pela população do ano e do período, respectivamente. Os municípios foram classificados quanto à transmissão de leishmaniose visceral, seguindo as recomendações da Superintendência de Controle de Endemias: transmissão esporádica (média anual de casos <2,4), moderada (média anual de casos ≥2,4 e <4,4) e intensa (média anual de casos ≥4,4) ^7^.

O risco de transmissão de leishmaniose visceral foi estimado pela divisão dos municípios em bairros, conforme a metodologia da Estratégia de Saúde da Família, para mitigar potencial viés de seleção e aumentar a representatividade municipal. A classificação do risco considerou a frequência de casos no período 2019-2022, a taxa de incidência e a razão cães/habitante. A frequência de casos em cada bairro foi classificada como baixa (1-2 anos), média (3 anos) ou alta (>4 anos), e a taxa de incidência, como baixa (inferior à taxa de incidência média acumulada) ou alta (superior à taxa de incidência média acumulada). A razão cães/habitante foi estimada pelos censos canino e humano realizados nesses bairros. 

A definição da amostra de quarteirões e imóveis, baseada no universo de imóveis e quarteirões (ativos e inativos) por bairro fornecido pelo Sistema de Cadastro de Localidade, empregou uma metodologia adaptada do Levantamento Rápido de Índices do *Aedes aegypti*^8^, com o objetivo de maximizar a precisão da estimativa dentro das restrições de custo. Em cada quarteirão sorteado, 50,0% dos domicílios foram sistematicamente visitados em ordem horária (primeiro sim, segundo não, terceiro sim, e subsequentes em caso de fechamento) até completar a amostra. 

Para analisar as vulnerabilidades de saúde na transmissão da leishmaniose visceral, estabeleceram-se três categorias (social, econômica e ambiental) e 25 quesitos (Q). A vulnerabilidade social foi avaliada por: ausência de pavimentação nas ruas (Q1), exposição a esgoto a céu aberto (Q2), acúmulo de lixo doméstico (Q3) ou de outros tipos de lixo (Q4), entulhos de terra e/ou matérias orgânicas (Q5), entulhos de madeiras, folhas etc. (Q6), terrenos baldios (Q7), circulação de cães errantes (Q8) e canis individuais ou coletivos (Q9). A vulnerabilidade econômica avaliou presença de domicílio de alvenaria (Q10), área externa do domicílio com reboco (Q11), presença de calçada (Q12), quintal de alvenaria (Q13), sem muro ou com cerca de madeira (Q14), piso do quintal de terra (Q15), lixo ou entulhos no quintal (Q16), umidade no quintal (Q17), plantas no quintal (Q18). A vulnerabilidade ambiental abrangeu a presença de vegetação alta (Q19) ou rasteira (Q20), de lagos ou lagoas de água (Q21), umidade decorrente da falta de saneamento básico (Q22), criação de animais de produção (Q23), galinheiros (Q24) e chiqueiros ou currais (Q25). 

Para mitigar a subjetividade na avaliação das vulnerabilidades de saúde, foram desenvolvidos checklists estruturados com indicadores definidos para cada variável. A presença ou ausência de cada indicador foi registrada, padronizando a observação. Também se documentou fotograficamente uma amostra das condições observadas.

Processamento e análise dos dados

Os dados foram processados e tabulados por meio dos softwares TabWin32 e Microsoft Excel 365. A análise estatística incluiu o cálculo de distribuições absolutas e relativas, bem como a aplicação da análise de correspondência para investigar associações entre municípios/bairros e variáveis de vulnerabilidade. A regressão logística binária foi aplicada para estimar a associação entre o município de residência (Salgueiro vs. Mirandiba) e a probabilidade de transmissão da leishmaniose visceral canina. Como variável dependente, considerou-se a positividade canina (1=positivo; 0=negativo), e, como variável explicativa, o município (1=Salgueiro; 0=Mirandiba). Todas as análises estatísticas foram conduzidas no software R versão 4.3.3, com o suporte dos pacotes FactoMineR e Factoextra ^9^^,^^10^.

## Resultados 

Entre janeiro de 2013 e dezembro de 2022, foram notificados 93 casos novos de leishmaniose visceral em Mirandiba e Salgueiro. Destes, 26,8% (n=25) ocorreram em Mirandiba, e 73,2% (n=68), em Salgueiro. A média anual de casos foi 2,5 em Mirandiba e 6,8 em Salgueiro. As taxas de incidência variaram de 0-53,0 casos por 100 mil habitantes em Mirandiba e de 1,6-26,9 casos por 100 mil habitantes em Salgueiro. As maiores taxas de incidência em Mirandiba foram observadas em 2015 (53,0) e 2017 ^26^^,^^2^ e em Salgueiro em 2014 ^26^^,^^9^ e 2017 ^21^^,^^5^ (Figura 1). Embora Salgueiro tenha apresentado o maior número de casos, a taxa de incidência acumulada no período foi superior em Mirandiba (16,4 vs. 11,2).

**Figura 1:** Total de casos e taxa de incidência por 100 mil habitantes de leishmaniose visceral. Mirandiba e Salgueiro, 2013-2022

A leishmaniose visceral apresentou características sociodemográficas semelhantes nos dois municípios, com maior frequência no sexo masculino (n=18 em Mirandiba e n=43 em Salgueiro), correspondendo a 72,0% e 63,0% dos casos. A faixa etária mais acometida foi de 1-4 anos (n=6 em Mirandiba e n=18 em Salgueiro) e representou 24,0% e 27,0% dos casos.

Em Mirandiba, 100,0% dos bairros listados na Estratégia de Saúde da Família (n=2: Cohab e Projetada) apresentaram casos de leishmaniose visceral no período 2019-2022 e foram incluídos na análise de risco. Em Salgueiro, dos 14 bairros listados foram incluídos apenas 29,0% (n=4: Barriguda, Divino, Planalto e Prado), responsáveis por 50,0% dos casos notificados no período (n=7/14). A frequência de casos em anos consecutivos foi considerada baixa (1-2 casos) em todos os bairros, classificando-os como de baixo risco de transmissão. No entanto, Prado e Cohab foram classificados como de alto risco com base na taxa de incidência média acumulada, superior à taxa de incidência geral (Tabela 1). 

Em Mirandiba, foram selecionados 15 quarteirões e visitados 217 domicílios, distribuídos entre Cohab (8 quarteirões e 112 domicílios) e Projetada (7 quarteirões e 105 domicílios). No bairro Cohab, a razão cães/habitante foi 0,15 (52 cães para 353 habitantes), enquanto no Projetada foi 0,14 (49 cães para 360 habitantes). Em Salgueiro, foram selecionados 20 quarteirões e visitados 240 domicílios, com a seguinte distribuição por bairro: Barriguda (2 quarteirões e 30 domicílios), Divino (3 quarteirões e 63 domicílios), Planalto (12 quarteirões e 47 domicílios) e Prado (3 quarteirões e 100 domicílios). A razão cães/habitante foi 0,14 (16 cães para 117 habitantes) em Barriguda, 0,15 (29 cães para 199 habitantes) em Divino, 0,21 (36 cães para 174 habitantes) em Planalto e 0,14 (42 cães para 293 habitantes) em Prado. Observaram-se a ordem de prioridade para ações de controle, estabelecida com base na classificação de risco (Tabela 1), e a distribuição espacial das áreas de risco nos dois municípios (Figura 2).

**Tabela 1:** Ordem de prioridade das ações de controle com base na frequência de casos, taxa de incidência (TI) por 100 mil habitantes e razão cães/habitante (RCH). Mirandiba e Salgueiro, 2019-2022

Município	Bairro	Frequência de casos	CFC^a^	População de 2022	TI	IMAc^b^	CTI^c^	RCH	Ordem de prioridade
Mirandiba	Cohab	1	Baixa	3.153	7,90	6,55	Alta	0,15	1
	Projetada	1	Baixa	4.815	5,20	-	Baixa	0,14	2
	Total	2	-	7.968	-	-	-	0,14	-
Salgueiro	Barriguda	1	Baixa	2.298	10,90	11,72	Baixa	0,14	4
	Divino	2	Baixa	5.042	9,90	-	Baixa	0,15	3
	Planalto	2	Baixa	4.663	10,70	-	Baixa	0,21	2
	Prado	2	Baixa	3.253	15,40	-	Alta	0,14	1
	Total	7	-	15.256	-	-	-	0,16	-

a Classificação de acordo com a frequência de casos; ^b^Taxa de incidência média acumulada por 100 mil habitantes; ^c^Classificação de acordo com a taxa de incidência.

**Figura 2:** Distribuição espacial das áreas de risco de transmissão de leishmaniose visceral por bairro. Salgueiro (A) e Mirandiba (B), 2022 ^a^Prefeitura municipal; ^b^Ordem de prioridade das ações de controle (1 - maior e 4 - menor).

**Figura 3:** Análise de correspondência para as vulnerabilidades: (A) sociais (Q1-Q9), (B) econômicas (Q10-Q18) e (C) ambientais (Q19-Q25) nos bairros dos municípios estudados. Mirandiba (azul) e Salgueiro (vermelho), 2022 ^Q1^Ausência de pavimentação nas ruas, ^Q2^Exposição a esgoto a céu aberto, ^Q3^Acúmulo de lixo doméstico, ^Q4^Acúmulo de outros tipos de lixo, ^Q5^Presença de entulhos de terra e/ou matérias orgânicas, ^Q6^Entulhos de madeiras, folhas etc., ^Q7^Terreno baldio, ^Q8^Circulação de cães errantes, ^Q9^Canis individuais ou coletivos, ^Q10^Domicílio de alvenaria, ^Q11^Área externa do domicílio com reboco, ^Q12^Presença de calçada, ^Q13^Quintal de alvenaria, ^Q14^Quintal sem muro ou com cerca de madeira, ^Q15^Piso do quintal de terra, ^Q16^Lixo ou entulhos no quintal, ^Q17^Umidade no quintal, ^Q18^Plantas no quintal, ^Q19^Presença de vegetação alta, ^Q20^Presença de vegetação rasteira, ^Q21^Lagos ou lagoas de água, ^Q22^Umidade decorrente da falta de saneamento básico, ^Q23^Criação de animais de produção, ^Q24^Galinheiros e ^Q25^Chiqueiros ou currais.

A análise de correspondência sugeriu que existiam diferenças nos padrões de vulnerabilidades social, econômica e ambiental entre os bairros de Salgueiro e Mirandiba, pois foram observadas diferentes associações em cada bairro (Figura 3). As vulnerabilidades apresentaram-se relativamente dispersas, indicando que diferentes bairros podem estar associados a diferentes conjuntos de vulnerabilidades ou a um conjunto único de vulnerabilidades.

O gráfico de vulnerabilidades sociais (Figura 3A) apresentou duas dimensões: a dimensão 1 explicou 40,8% variância total, e a dimensão 2, 35,0% da variância total. Em Mirandiba, as vulnerabilidades foram associadas à presença de entulhos (Q5) no Cohab. No bairro Projetada, as vulnerabilidades foram quanto a ruas não pavimentadas (Q1), presença de lixo doméstico (Q3) e outros tipos de lixo (Q4), cães errantes (Q8) e canis individuais ou coletivos (Q9). Em Salgueiro, não houve associação significativa em Barriguda. No entanto, Divino associou-se à presença de entulhos de madeiras e folhas (Q6) e cães errantes (Q8); Planalto, à presença de outros tipos de lixo (Q4) e terrenos baldios (Q7) e à circulação de cães errantes (Q8); e Prado, ao acúmulo de lixo doméstico (Q3) e à circulação de cães errantes (Q8). 

No gráfico de vulnerabilidades econômicas (Figura 3B), a dimensão 1 explicou 52,0% variância total, e a dimensão 2, 30,1% desta. Em Mirandiba, as vulnerabilidades foram associadas à presença de quintal com piso de terra (Q15) no bairro Cohab e área externa do domicílio com reboco (Q11) no Projetada. Em Salgueiro, o bairro Barriguda associou-se à presença de lixo ou entulhos no quintal (Q16); no Divino, à área externa do domicílio com reboco (Q11), quintal sem muro ou com cerca de madeira (Q14) e piso de terra (Q15); no Planalto, a quintais de alvenaria (Q13) e quintais com presença de plantas (Q18); e no Prado, à área externa do domicílio com reboco (Q11) e quintal sem muro ou com cerca de madeira (Q14).

No gráfico de vulnerabilidades ambientais (Figura 3C), as dimensões 1 e 2 explicaram 65,9% e 20,2% da variância total. Em Mirandiba, as vulnerabilidades foram associadas à presença de vegetação alta (Q19) nos bairros Cohab e Projetada, que também apresentou associação com vegetação rasteira (Q20). Em Salgueiro, o bairro Barriguda associou-se a galinheiros (Q24); Divino e Prado, à presença de lagos ou lagoas (Q21), umidade por falta de saneamento (Q22) e presença de criação de animais de produção (Q23); e Planalto, à criação de animais de produção (Q23) e presença de chiqueiros ou currais (Q25). 

A prevalência de leishmaniose visceral canina foi significativamente maior (p-valor=0,99) em Salgueiro (21,4%) do que em Mirandiba (9,5%). Em Salgueiro, a positividade variou de 6,0%-55,0%, e a negatividade, de 45,0%-94,0%. A maior positividade foi em 2015, e a maior negatividade, em 2018. Em Mirandiba, a positividade variou de 0,0%-49,0%, e a negatividade, de 0,0%-100,0%. A maior positividade foi em 2015, e maior negatividade, em 2013 e 2020. 

O modelo logístico, que utilizou Mirandiba como categoria de referência (*odds ratio*, OR 1,0), mostrou que cães residentes em Salgueiro tiveram uma chance duas vezes maior de serem soropositivos para leishmaniose visceral em comparação aos cães de Mirandiba (OR 2,00; intervalo de confiança de 95% 1,32; 3,03; p-valor=0,001). Este achado indicou que, mesmo controlando a variabilidade temporal, Salgueiro apresentou risco significativamente superior, o que justifica sua classificação como área prioritária para ações de vigilância e controle. 

O gráfico de tendência (Figura 4) demonstrou que, apesar da redução nas taxas ao longo dos anos, a probabilidade estimada de positividade canina permaneceu consistentemente mais elevada em Salgueiro. Os intervalos de confiança destacados na curva (áreas sombreadas em verde) reforçaram a robustez da estimativa.

**Figura 4:** Prevalência de leishmaniose visceral canina. Salgueiro e Mirandiba, 2013-2022

## Discussão 

A leishmaniose visceral continua sendo um problema de saúde pública em Mirandiba e Salgueiro, ambos classificados como áreas de transmissão intensa ^7^, com incidências superiores às médias nacional e estadual ^11^^,^^12^, mesmo após a tendência de declínio observada a partir de 2017. Os achados demonstram que a persistência da transmissão está vinculada a fatores estruturais, como pobreza, saneamento deficiente, descarte inadequado de resíduos sólidos ^13^, urbanização não planejada ^11^ e elevada prevalência de reservatórios caninos infectados ^14^^,^^15^.

A maior ocorrência de leishmaniose visceral em indivíduos do sexo masculino, compatível com achados anteriores ^5^^,^^16^, pode estar relacionada a fatores hormonais, maior exposição ocupacional e padrões de mobilidade que coincidem com os horários de atividade alimentar do vetor ^16^. Esse perfil é especialmente relevante em regiões onde a agricultura predomina como principal fonte de renda ^12^, como nos municípios estudados.

A elevada incidência em crianças de 1-4 anos, também observada em outros estudos ^5^^,^^11^^,^^17^, destaca o papel da transmissão no ambiente doméstico e peridomiciliar. A suscetibilidade imunológica associada à imaturidade do sistema imunológico e à presença de deficiências nutricionais ^18^, somada à interação com animais domésticos ^19^, contribui para o risco aumentado nesse grupo etário. Esses achados reforçam a necessidade de estratégias intersetoriais voltadas à melhoria do estado nutricional ^18^, ao controle populacional canino e à vigilância ativa.

A análise multivariada permitiu identificar perfis distintos de vulnerabilidades territoriais, como acúmulo de lixo, presença de entulhos, vegetação densa, criação de animais e cães errantes ^20^^,^^21^^,^^22^^,^^23^. Tais achados contribuem para o entendimento da dinâmica de urbanização da doença e subsidiam o planejamento territorial de ações de vigilância e controle.

A priorização dos territórios para controle da leishmaniose visceral foi realizada com base em múltiplos critérios epidemiológicos e ambientais, incluindo frequência de casos humanos, taxa de incidência média acumulada, proporção de bairros afetados, razão cães/habitante e prevalência de cães soropositivos - abordagem amplamente recomendada para delimitação de áreas de risco em cenários de transmissão urbana da doença ^5^^,^^17^. A aplicação integrada desses indicadores permitiu classificar os territórios estudados em áreas de baixo, médio e alto riscos, orientando ações de vigilância e controle mais direcionadas. 

Por exemplo, em Mirandiba, o bairro Cohab apresentou incidência acumulada superior à média municipal, razão cães/habitante acima de 0,14 e presença de múltiplas vulnerabilidades estruturais, o que justifica sua inclusão como área de prioridade elevada. Em Salgueiro, os bairros Prado e Planalto concentraram fatores de risco epidemiológicos e ambientais simultâneos, como elevada prevalência canina, circulação de cães errantes e vulnerabilidades ambientais (terrenos baldios, lixo acumulado e criação de animais de produção), elementos que favorecem a manutenção de ecótopos vetoriais urbanos ^22^^,^^23^^,^^24^. 

A alta prevalência de leishmaniose visceral canina em Salgueiro (21,4%), aliada à presença de cães soropositivos assintomáticos com capacidade de infectar o vetor ^25^^,^^26^, resultou em risco estimado 100,0% superior ao de Mirandiba. Isso reforçou a importância da combinação de parâmetros epidemiológicos e ambientais para definição territorial ^5^.

A análise de correspondência confirmou que as vulnerabilidades não estão homogeneamente distribuídas, variando entre bairros e municípios. Em Mirandiba, observou-se associação com ruas não pavimentadas, lixo doméstico, quintais com acúmulo de resíduos e presença de canis improvisados - condições que favorecem a proliferação de flebotomíneos ^24^^,^^25^^,^^26^. Em Salgueiro, destacou-se a proximidade de galinheiros, lagoas, terrenos baldios e áreas úmidas por ausência de saneamento básico, fatores diretamente associados ao aumento da densidade vetorial ^14^^,^^22^^,^^23^. 

Esses achados reforçam que a priorização territorial deve adotar modelo multidimensional, incorporando fatores epidemiológicos, sociais, ambientais e estruturais, conforme diretrizes estabelecidas ^27^ e evidências científicas ^5^^,^^22^^,^^27^. Essa abordagem contribui para a alocação eficiente de recursos, o planejamento de ações integradas e o fortalecimento das políticas públicas locais.

Embora os achados deste estudo sejam relevantes, algumas limitações metodológicas devem ser consideradas. A seleção dos bairros foi baseada em critérios operacionais e logísticos, com inclusão restrita a áreas cobertas por equipes da Estratégia de Saúde da Família e com histórico de notificações, o que pode ter comprometido a representatividade territorial e gerado superestimação ou subestimação do risco em determinadas localidades. A ausência de sorteio aleatório e de amostragem probabilística limitou a extrapolação dos resultados para os demais bairros dos municípios. A subnotificação de casos humanos durante a pandemia de covid-19, particularmente em 2020 e 2021, também impactou a análise temporal, reduzindo a acurácia das estimativas de incidência e dificultando a identificação de tendências epidemiológicas ^17^. A ausência de dados sobre densidade vetorial impediu correlações mais robustas entre os fatores ambientais e a dinâmica de transmissão, restringindo a compreensão do papel dos flebotomíneos na manutenção do ciclo da doença. 

Essas limitações reforçam a necessidade de estudos futuros com maior abrangência territorial, monitoramento entomológico sistemático e avaliação dos efeitos da pandemia sobre os indicadores de vigilância. Ainda assim, os resultados apresentados oferecem subsídios relevantes para o planejamento municipal, especialmente no que se refere à delimitação de áreas prioritárias.

Recomendam-se estratégias integradas e sustentáveis que articulem saúde, meio ambiente, infraestrutura e educação, voltadas ao enfrentamento da leishmaniose visceral em contextos urbanos e vulneráveis. A adoção de critérios territoriais no planejamento permite direcionar recursos conforme o risco epidemiológico e ambiental, com ações focalizadas em bairros prioritários, como os identificados neste estudo. 

Além da vigilância epidemiológica e entomológica, é fundamental fortalecer o diagnóstico precoce, o monitoramento da população canina com medidas sanitárias adequadas e o controle ambiental de criadouros vetoriais. A participação comunitária deve ser incentivada por meio de educação em saúde, campanhas de conscientização sobre o manejo de resíduos e convivência segura com animais domésticos. Tais ações devem ser contínuas e articuladas com políticas de saneamento, proteção social e desenvolvimento urbano sustentável.

Os resultados obtidos oferecem suporte técnico à formulação de políticas públicas territorializadas, equitativas e baseadas em evidências, fundamentais para reduzir a carga da leishmaniose visceral e fortalecer o controle em municípios com elevada vulnerabilidade socioambiental.

## Data Availability

Os dados estão disponíveis em: https://zenodo.org/records/15933982.
